# Novel Results on SNR Estimation for Bandlimited Optical Intensity Channels

**DOI:** 10.3390/s24010023

**Published:** 2023-12-19

**Authors:** Wilfried Gappmair

**Affiliations:** Institute of Communication Networks and Satellite Communications, Graz University of Technology, Inffeldgasse 12, 8010 Graz, Austria; gappmair@tugraz.at

**Keywords:** SNR estimation, optical wireless communications, intensity modulation

## Abstract

In a previous work of the author about non-data-aided estimation of the signal-to-noise ratio (SNR) for bandlimited optical intensity channels, a couple of limitations have been identified in terms of error performance and computational complexity. In the current paper, these deficiencies are avoided by the introduction of a second receiver filter with specific properties that is operated in parallel to the receiver filter normally used in this respect. Although not initially intended, the concept is also applied to data-aided SNR estimation by deriving a maximum likelihood algorithm and the Cramer–Rao lower bound (CRLB) as the theoretical limit of the error performance. In the next step, the dual-filter framework is used in the context of SNR estimation without knowledge about data symbols. The most significant benefit of this method is that the number of payload data employed for the estimation procedure might be selected arbitrarily long without impacting the spectral efficiency of the link. Since the computation of the true CRLB was out of scope due to complexity reasons, an asymptotic variant for very low SNR values is analyzed, which ends up in a closed-form solution. Furthermore, an algorithm based on first- and second-order moments of the samples at the dual-filter output is investigated, which turned out to be very attractive in terms of error performance and computational complexity.

## 1. Introduction

There is no doubt that optical wireless communication (OWC) solutions have a lot of benefits compared to their radio frequency (RF) counterparts: rather inexpensive and easy to deploy, extremely high throughput, no problems with data security, no regulatory and license issues, just to mention the most significant aspects in this context [1,2,3,4]. However, when it comes to optical intensity modulation, a unipolar signal concept with respect to symbol constellation and pulse shaping is of paramount importance. Realized via pulse amplitude modulation (PAM) and root-raised cosines, normally used in the RF domain for pulse shaping, this has been investigated in [5,6] by assuming an appropriately selected bias. Although the approach is strictly bandlimited, the price to be paid is some clipping effect and less efficiency in terms of power and energy.

As an alternative, squared raised cosine and double jump functions have been suggested in [7] for pulse shaping in bandlimited optical intensity links. Clipping as well as bias problems are completely avoided by this method, with the additional benefit that the Nyquist property is still satisfied, which allows a simple detection procedure without the introduction of inter-symbol interference effects.

Nevertheless, even for OWC systems, the most important transmission parameters must be recovered at the receiver by suitable algorithms. In this context, some knowledge about the signal-to-noise ratio (SNR) is indispensable for a lot of scenarios; e.g., many adaptive systems [8] or powerful error correction schemes [9] necessitate this sort of information so that the link can be operated close to the Shannon bound. In this context, a non-data-aided (NDA) algorithm for SNR estimation has been discussed by the author in a recently published paper [10], i.e., no a priori knowledge about data is needed in the receiver unit for this purpose, or, in other words, payload data might be used so that the spectral efficiency does not depend on the observation length required for the estimation process. This is in striking contrast to a data-aided (DA) approach, where part of the data frame is occupied by pilot sequences [11].

However, the solution developed in [10] is based on the expectation-maximization (EM) principle [12,13,14], which turned out to be the only way to approach the Cramer–Rao lower bound (CRLB) as the theoretical limit of the error performance [15,16,17]. Other algorithms for SNR estimation have been studied as well, e.g., moment-based or decision-directed methods, but they failed insofar as they exhibit a non-negligible bias and/or jitter effect. Unfortunately, the computational load of EM algorithms is considerable, so a less complex SNR estimator would be most welcome. In the current paper, this is realized by the introduction of a second filter operated in parallel to the receiver filter and using both outputs in a moment-based approach, which is characterized by its computational simplicity and the fact that the performance degradation of EM solutions in the medium SNR range is avoided.

The rest of the paper is organized as follows: In Section 2, the signal and channel model is introduced, which we use for analytical and simulation work in the sequel. In Section 3, a maximum likelihood algorithm is investigated for DA SNR estimation, complemented by the derivation of the related CRLB as the theoretical limit of the error performance. The focus of Section 4 is on the development of a method based on the first- and second-order moments of the samples available at the output of the receiver filters. Since the computation of the true CRLB is out of scope in the context of NDA SNR estimation, we concentrate on an asymptotic variant, which is available in closed form and applies to smaller SNR values. Numerical results related to bias effect and error performance are presented in Section 5. Finally, conclusions are drawn in Section 6.

## 2. Signal and Channel Model

The signal and channel model for the current paper are more or less the same as those in [10,11] for NDA as well as DA estimation of the SNR, respectively. Nevertheless, for convenience, the model is briefly recapitulated in the sequel. On top of that, it is also extended in a suitable manner so that new results are achievable.

In the following, it is assumed that the data symbols *a_k_*, k∈Z, are independent and identically distributed (i.i.d.) elements of an *M*-ary PAM alphabet A. In this context, it makes sense to normalize the symbols such that $E[a_{k}^{2}]=1$, where E[·] denotes the expectation operator. This is straightforwardly achieved by $A=\frac{1}{\sqrt{\eta_{M}}}\{0,\;\;1,\;\;\ldots ,\;\;M-1\}$ and $\eta_{M}=\frac{1}{6}(M-1)(2M-1)$. As a consequence, the average symbol value is given by

(1) $$\mu_{a}=E[a_{k}]=\frac{1}{\sqrt{\eta_{M}}}\;\;\frac{M-1}{2}=\sqrt{\frac{3\;(M-1)}{2\;(2M-1)}}$$

If we adopt a pulse shape expressed by *h*(*t*), the signal at the output of the opto-electrical receiver module develops as [10,11]

(2) $$r(t)=A\;\sum_{k}a_{k\;}h(t-kT-\tau )+w(t)$$

where *A* > 0 is the channel gain, *T* and *τ* specify the symbol period as well as the propagation delay between transmitter and receiver, respectively. Of course, the signal part in (2) is impaired by *w*(*t*), representing a zero-mean white Gaussian noise component with variance $\sigma_{w}^{2}$.

Furthermore, by introduction of

(3) $$\bar{h}=\frac{1}{\sqrt{T}}\int_{-\infty }^{\infty }h(t)\;dt$$

and the average optical power $P_{0}=\mu_{a}\bar{h}$, the average electrical SNR is described as

(4) $$\gamma_{s}=\frac{A^{2}P_{0}^{2}}{\sigma_{w}^{2}}$$

**Figure 1:**
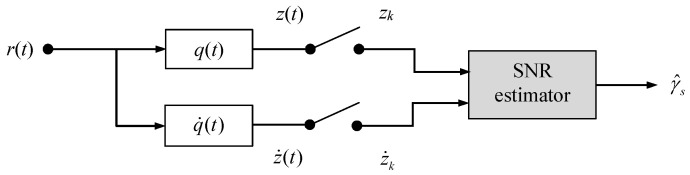
Signal model for SNR estimation using a dual-filter framework.

Before the signal in (2) can be processed in further receiver stages, it has to be filtered appropriately. Denoting the corresponding impulse response by *q*(*t*), the related output is obtained as z(t)= q(t)⊗r(t), where ⊗ characterizes the convolutional operator. This scenario is basically illustrated in Figure 1. Regarding a radio frequency (RF) system, *q*(*t*) would be designed as a matched filter in order to maximize the SNR at the output. It has been shown in [18,19] that suitable pairs of transmitter-receiver filters satisfying the non-negativity and Nyquist properties are only attainable via an unconstrained min–max optimization procedure, which must be solved by rather cumbersome numerical means whenever a new filter design is required. In order to avoid this, it is suggested to implement a rectangular shape over the spectrum occupied by the user component in (2).

By application of the Fourier transform [20], we simply obtain $Q(f)=F[q(t)]=\sqrt{T}$ for |f|  ≤(1+α)/T and Q(f)=0 elsewhere, with *α* as the roll-off factor (excess bandwidth) of the selected pulse shape. As a result, the signal parts of *r*(*t*) and *z*(*t*) are the same, whereas the noise component is determined by *n*(*t*) = *w*(*t*) ⊗ *q*(*t*), representing a zero-mean non-white Gaussian process.

As already mentioned previously, a second filter will be operated in parallel to the receiver filter, as visualized in Figure 1. Performing an impulse response expressed by $\dot{q}(t)$, the corresponding output is given by $\dot{z}(t)=\;\dot{q}(t)⊗r(t)$. Under the assumption that the symbol timing has been reliably recovered by properly selected estimation or synchronization algorithms [21,22,23], it is required that $\dot{q}(t)$ exhibits an impulse response such that the signal component of $\dot{z}(t)$ vanishes at integer multiples of the symbol period, which is satisfied only if ${\left.h(t)⊗\dot{q}(t)\right|}_{t=kT}=0$ for all k∈Z. In this respect, it is to be noticed that the first-order derivative of squared raised cosine or double jump functions fulfills this pre-requisite [7], i.e., ${\left.\partial h(t)/\partial t\right|}_{t=kT}=0,\;\;\;k\in Z$. Hence, by shifting this problem to the frequency domain, the Fourier transform of $\dot{q}(t)$ is determined by $\dot{Q}(f)=\;F[\dot{q}(t)]=$j2πfT Q(f).

After having synchronized symbol timing and clocking, e.g., by implementation of one of the recovery algorithms investigated in [21,22,23], the *T*-spaced samples at the output of the dual-filter concept are furnished by

(5) $$z_{k}=z(kT)=A\cdot a_{k}+n_{k\;}$$

and

(6) $${\dot{z}}_{k}=\dot{z}(kT)={\dot{n}}_{k\;}$$

It is to be emphasized that the parallel filter delivers solely noise samples denoted by ${\dot{n}}_{k}$, which is explained by the filter properties introduced previously. Furthermore, both noise processes, $n_{k}$ and ${\dot{n}}_{k}$, have a zero-mean non-white Gaussian character.

## 3. Data-Aided SNR Estimation

### 3.1. Log-Likelihood Function

The Cramer–Rao lower bound (CRLB) is of paramount importance when it comes to the estimation of a parameter. This is not only true for communication links, as exemplified in [16,17], but relates to any technical system [15]. The reason behind this is the fact that it represents the theoretical limit of the error performance of any estimator developed in this respect. In order to derive the CRLB of SNR estimates achieved by the dual-filter framework in Figure 1, we assume that *L* observables $z_{k}$ and ${\dot{z}}_{k}$, *k* = 0, 1,…, *L* − 1 are available at the output of the receiver filters. Arranging these observables in vector form, we have that z=A·a+n and $\dot{z}=\dot{n}$, where a, n and $\dot{n}$ denote the corresponding data and noise sequences, each of them with *L* elements. For convenient reasons, we put these pieces together so that we have a single vector equation given by

(7) $$y=\left(\begin{array}{c} z\\ \dot{z} \end{array}\right)=A\cdot \left(\begin{array}{c} a\\ 0 \end{array}\right)+\left(\begin{array}{c} n\\ \dot{n} \end{array}\right)=A\cdot c+\nu$$

where **0** stands for a vector with *L* zero entries.

According to the channel model introduced in the previous section, the average electrical SNR in (4) is a function of the channel gain *A* and the standard deviation $\sigma_{w}$ of the zero-mean white Gaussian noise process. Since $P_{0}$ depends on the PAM scheme as well as the pulse shape selected for this purpose, both of which are known in advance by the receiver unit, we better focus on the estimation of the SNR normalized by $P_{0}^{2}$, i.e., $\rho_{s}=\gamma_{s}/P_{0}^{2}=A^{2}/\sigma_{w}^{2}$. Therefore, the parameter vector used for the computation of the CRLB is characterized by $u=(A,\;\sigma_{w})$.

The major ingredient for the derivation of the CRLB is knowledge of the likelihood function Pr(y|c;u) describing the signal model in (7). Since both noise components, n and $\dot{n}$, are zero-mean Gaussian variates, the likelihood function is furnished by [24,25]

(8) $$\mathrm{Pr}(y|c;u)=\frac{1}{\sqrt{{\left(2\pi \right)}^{2L}\det(R)}}e^{-\frac{1}{2}\;\;{\left(y-A\;c\right)}^{T}R^{-1}(y-A\;c)}$$

with $R=E[(y-A\;c){\left(y-Ac\right)}^{T}]$ as the corresponding covariance matrix. This 2*L* × 2*L* matrix can be partitioned into four *L* × *L* submatrices as follows:

(9) $$R=E[\nu \cdot \nu^{T}]=\left(\begin{array}{cc} E[n\cdot n^{T}] & E[n\cdot {\dot{n}}^{T}]\\ E[\dot{n}\cdot n^{T}] & E[\dot{n}\cdot {\dot{n}}^{T}] \end{array}\right)=\left(\begin{array}{cc} R_{11} & R_{12}\\ R_{21} & R_{22} \end{array}\right)$$

For line *i* = 0, 1,…, *L* − 1 and column *k* = 0, 1,…, *L* − 1, the entries of $R_{11}$ are determined by the auto-correlation of the noise samples $n_{i}$, i.e.,

(10) $$\begin{array}{cc} E[n_{i\;}n_{k}] & =\sigma_{w}^{2}\int_{-(1+\alpha )/T}^{\left(1+\alpha \right)/T}e^{j2\pi (i-k)fT}Q(f)Q(-f)\;df\\ & =2(1+\alpha )\;\sigma_{w}^{2}\;\mathrm{sinc}[2(1+\alpha )(i-k)] \end{array}$$

with sinc(x)=sin(πx)/(πx). On the other hand, the entries of $R_{12}$ characterize the cross-correlation between noise samples $n_{i}$ and ${\dot{n}}_{k}$, which is given by

(11) $$\begin{array}{ll} E[n_{i\;}{\dot{n}}_{k}] & =\sigma_{w}^{2}\int_{-(1+\alpha )/T}^{\left(1+\alpha \right)/T}e^{j2\pi (i-k)fT}Q(f)\dot{Q}(-f)\;df\\ & =2(1+\alpha )\sigma_{w}^{2}\left\{\begin{array}{l} 0,\;\;\;i=k\\ \frac{-\cos[2\pi (1+\alpha )(i-k)]+\mathrm{sinc}[2(1+\alpha )(i-k)]}{i-k},\;\;\;i\neq k \end{array}\right. \end{array}$$

The elements of $R_{21}$ are determined by $E[{\dot{n}}_{i}n_{k}]$, which is equivalent to (11) after having swapped indexes *i* and *k*, i.e., $R_{21}$ is the transpose of $R_{12}$. Finally, we have that the entries of $R_{22}$ are specified by the auto-correlation of ${\dot{n}}_{i}$, which yields

(12) $$\begin{array}{ll} E[{\dot{n}}_{i\;}{\dot{n}}_{k}] & =\sigma_{w}^{2}\int_{-(1+\alpha )/T}^{\left(1+\alpha \right)/T}e^{j2\pi (i-k)fT}\dot{Q}(f)\dot{Q}(-f)\;df\\ & =2(1+\alpha )\sigma_{w}^{2}\left\{\begin{array}{l} \frac{4\pi^{2}{\left(1+\alpha \right)}^{2}}{3},\;\;\;i=k\\ \frac{2\cos[2\pi (1+\alpha )(i-k)]+({\left[2\pi (1+\alpha )(i-k)\right]}^{2}-2)\mathrm{sinc}[2(1+\alpha )(i-k)]}{{\left(i-k\right)}^{2}},\;\;\;i\neq k \end{array}\right. \end{array}$$

Extracting the common factor, we simply obtain

(13) $$R=2(1+\alpha )\;\sigma_{w}^{2}\;\Omega$$

where the entries of **Ω** are only functions of the roll-off factor *α*. Furthermore, it is not difficult to show that $\Omega =\Omega^{T}$. Hence, with $\Psi =\Omega^{-1}$, $\Psi =\Psi^{T}$, and substituting (13) into (8), the log-likelihood function (LLF) of our signal model, i.e., Λ(y|c;u)=logPr(y|c;u), is expressed by

(14) $$\Lambda (y|c;u)=-L\log\sigma_{w}^{2}-\frac{y^{T}\Psi \;y-2A\;y^{T}\Psi \;c+A^{2}c^{T}\Psi \;c}{4(1+\alpha )\sigma_{w}^{2}}$$

after having omitted immaterial constants and factors not depending on **u**.

### 3.2. Modified Cramer–Rao Lower Bound

Knowing the LLF, we are able to derive the elements of the Fisher information matrix (FIM), which are determined by the second-order derivatives of the LLF with respect to *A* and $\sigma_{w}$. However, by definition of $P_{n}=\sigma_{w}^{2}$ and replacing the channel gain *A* in (14) by $\sqrt{\rho_{s}P_{n}}$, the derivatives are related to $\rho_{s}$ and $P_{n}$. This approach has the advantage that the CRLB for the normalized SNR is directly obtained without the application of cumbersome transformation rules [15]. As a consequence, we have that

(15) $$\frac{\partial^{2}\Lambda (y|c;u)}{\partial \rho_{s}^{2}}=-\frac{y^{T}\Psi \;c}{8(1+\alpha )\sqrt{\rho_{s}^{3}P_{n}}}$$

(16) $$\frac{\partial^{2}\Lambda (y|c;u)}{\partial P_{n}^{2}}=\frac{L}{P_{n}^{2}}-\frac{y^{T}\Psi \;y}{2(1+\alpha )P_{n}^{3}}+\frac{3\sqrt{\rho_{s}}y^{T}\Psi \;c}{8(1+\alpha )\sqrt{P_{n}^{5}}}$$

(17) $$\frac{\partial^{2}\Lambda (y|c;u)}{\partial \rho_{s\;}\partial P_{n}}=\frac{\partial^{2}\Lambda (y|c;u)}{\partial P_{n\;}\partial \rho_{s}}=-\frac{y^{T}\Psi \;c}{8(1+\alpha )\sqrt{\rho_{s}^{3}P_{n}}}$$

Plugging now $y=A\;c+\nu =\sqrt{\rho_{s}P_{n}}\;c+\nu$ into (15)–(17) and averaging with respect to ν provides us with FIM elements depending on the selected pilot sequence **c**~**a**. In order to avoid this sort of restriction, we may extend the expected operation to **c** as well, which constitutes the so-called modified Cramer–Rao lower bound (MCRLB) [26,27,28]. By doing so, we get

(18) $$J_{11}=-E\left[\frac{\partial^{2}\Lambda (y|c;u)}{\partial \rho_{s}^{2}}\right]=\frac{1}{8(1+\alpha )\rho_{s}}E_{c}[c^{T}\Psi \;c]$$

(19) $$J_{22}=-E\left[\frac{\partial^{2}\Lambda (y|c;u)}{\partial P_{n}^{2}}\right]=-\frac{L}{P_{n}^{2}}+\frac{\rho_{s}}{8(1+\alpha )P_{n}^{2}}E_{c}[c^{T}\Psi \;c]+\frac{1}{2(1+\alpha )P_{n}^{3}}E_{\nu }[\nu^{T}\Psi \;\nu ]$$

(20) $$J_{12}=-E\left[\frac{\partial^{2}\Lambda (y|c;u)}{\partial \rho_{s}\partial P_{n}}\right]=\frac{1}{8(1+\alpha )P_{n}}E_{c}[c^{T}\Psi \;c]$$

As a result, the MCRLB for $\rho_{s}$ is achieved as

(21) $$\begin{array}{ll} \mathrm{MCRLB}(\rho_{s}) & =\frac{J_{22}}{J_{11}J_{22}-J_{12}^{2}}\\ & =2(1+\alpha )\rho_{s}^{2}\left(\frac{P_{n}}{E_{\nu }[\nu^{T}\Psi \;\nu ]-2L(1+\alpha )P_{n}}+\frac{4}{\rho_{s}\;E_{c}[c^{T}\Psi \;c]}\right) \end{array}$$

with $E_{c}[\cdot ]$ and $E_{\nu }[\cdot ]$ indicating the averaging procedures with respect to data and noise. Next, we assume that the 2*L* × 2*L* matrix **Ψ** is partitioned into four *L* × *L* submatrices according to

(22) $$\Psi =\left(\begin{array}{cc} \Psi_{11} & \Psi_{12}\\ \Psi_{21} & \Psi_{22} \end{array}\right)$$

where $\psi_{mn,ik}$ denotes the entry of $\Psi_{mn}$ for line *i* and column *k*. Hence, by considering the definition of **c** in (7), it is clear that

(23) $$E_{c}[c^{T}\Psi \;c]=E_{a}[a^{T}\Psi_{11}\;a]=\sum_{i=0}^{L-1}\sum_{k=0}^{L-1}E[a_{i}a_{k}]\;\psi_{11,ik}=L({\bar{\Psi }}_{0}+2\mu_{a}^{2}{\bar{\Psi }}_{1})$$

after having taken into account that $E[a_{i}^{2}]=1$ and $E{\left[a_{i}a_{k}\right]}_{i\neq k}=\mu_{a}^{2}$ as well as using the sums

(24) $${\bar{\Psi }}_{0}=\frac{1}{L}\sum_{i=0}^{L-1}\psi_{11,ii},\;\;\;{\bar{\Psi }}_{1}=\frac{1}{L}\sum_{i=0}^{L-1}\sum_{k=i+1}^{L-1}\psi_{11,ik}$$

Furthermore, if we employ the results in (10)–(12), the averaging procedure with respect to noise yields

(25) $$\begin{array}{ll} E_{\nu }[\nu^{T}\Psi \;\nu ] & =E_{\nu }[n^{T}\Psi_{11\;}n+n^{T}\Psi_{12\;}\dot{n}+{\dot{n}}^{T}\Psi_{21\;}n+{\dot{n}}^{T}\Psi_{22\;}\dot{n}]\\ & =\sum_{i=0}^{L-1}\sum_{k=0}^{L-1}\left\{E[n_{i}n_{k}]\;\psi_{11,ik}+E[n_{i}{\dot{n}}_{k}]\;\psi_{12,ik}+E[{\dot{n}}_{i}n_{k}]\;\psi_{21,ik}+E[{\dot{n}}_{i}{\dot{n}}_{k}]\;\psi_{22,ik}\right\}\\ & =4L(1+\alpha )\;P_{n}\;{\bar{\Psi }}_{2} \end{array}$$

where

(26) $${\bar{\Psi }}_{2}=\frac{1}{2L}\sum_{i=0}^{2L-1}\sum_{k=0}^{2L-1}\omega_{ik}\;\psi_{ik}$$

with $\omega_{ik}$ and $\psi_{ik}$ as the entries of **Ω** and **Ψ**, respectively. Finally, by putting all these pieces together, the MCRLB in (21), for convenient reasons normalized by $\rho_{s}^{2}$, boils down to

(27) $$\mathrm{NMCRLB}(\rho_{s})=\frac{\mathrm{MCRLB}(\rho_{s})}{\rho_{s}^{2}}=\frac{1}{L}\left(\frac{1}{2{\bar{\Psi }}_{2}-1}+\frac{8(1+\alpha )}{\rho_{s}({\bar{\Psi }}_{0}+2\mu_{a}^{2}{\bar{\Psi }}_{1})}\right)$$

### 3.3. Maximum Likelihood Estimator

The closed form of the LLF in (14) gives us the chance to develop a maximum likelihood (ML) estimator in a fairly straightforward manner by computing the first-order derivatives of (14) with respect to channel gain *A* and noise power *P_n_*, equating these results to zero, and solving both relationships for *A* and *P_n_*. Specifically, we have for **u** = (*A*, *P_n_*)

(28) $${\left.\frac{\partial \Lambda (y|c;u)}{\partial A}\right|}_{u=\hat{u}}=\frac{y^{T}\Psi \;c-\hat{A}\;\;c^{T}\Psi \;c}{2(1+\alpha ){\hat{P}}_{n}}=0$$

and

(29) $${\left.\frac{\partial \Lambda (y|c;u)}{\partial P_{n}}\right|}_{u=\hat{u}}=-\frac{L}{{\hat{P}}_{n}}+\frac{y^{T}\Psi \;y-2\hat{A}\;y^{T}\Psi \;c+{\hat{A}}^{2}c^{T}\Psi \;c}{4(1+\alpha ){\hat{P}}_{n}^{2}}=0$$

Introducing in the next step $M_{cc}=c^{T}\Psi \;c$, $M_{cy}=y^{T}\Psi \;c$, and $M_{yy}=y^{T}\Psi \;y$, the estimates for channel gain and noise power are furnished by

(30) $$\hat{A}=\frac{y^{T}\Psi \;c}{c^{T}\Psi \;c}=\frac{M_{cy}}{M_{cc}},\;\;\;M_{cc}>0$$

and

(31) $${\hat{P}}_{n}=\frac{y^{T}\Psi \;y-2\hat{A}\;y^{T}\Psi \;c+{\hat{A}}^{2}c^{T}\Psi \;c}{4(1+\alpha )L}=\frac{1}{4(1+\alpha )L}\left(M_{yy}-\frac{M_{cy}^{2}}{M_{cc}}\right)$$

According to the invariance principle [29,30], the ML estimate is then given by

(32) $${\hat{\rho }}_{s}=\frac{{\hat{A}}^{2}}{{\hat{P}}_{n}}$$

Note that *M_cc_* must be larger than zero; otherwise, the algorithm fails, i.e., a pilot sequence constituted by only zero elements would not work. In the following, the ML algorithm is summarized to be executed step by step (Algorithm 1):
**Algorithm 1**: ML estimator.      **Initialization**           Pre-calculation of
$\Psi \leftarrow \;\;\Omega^{-1}$           Creation of pilot sequence **c** and vector **y** of observables      **Computation**           Auxiliary terms:
$M_{cc}\leftarrow c^{T}\Psi \;c,\;\;M_{cy}\leftarrow y^{T}\Psi \;c,\;\;M_{yy}\leftarrow y^{T}\Psi \;y$           Channel gain estimate:
$\hat{A}\leftarrow \frac{M_{cy}}{M_{cc}}$           Noise power estimate: ${\hat{P}}_{n}\leftarrow \frac{1}{4(1+\alpha )L}\left(M_{yy}-\frac{M_{cy}^{2}}{M_{cc}}\right)$      **Output**           SNR estimate:
${\hat{\rho }}_{s}\leftarrow \frac{{\hat{A}}^{2}}{{\hat{P}}_{n}}$

Since **Ψ** is a 2*L* × 2*L* matrix and **c** as well as **y** are vectors with 2*L* entries each, it is clear that the computational complexity of the SNR estimate is in the order of $O(L^{2})$ real-valued additions and multiplications.

## 4. Non-Data-Aided SNR Estimation

### 4.1. Asymptotic Cramer–Rao Lower Bound

For NDA estimation of the SNR, we cannot assume that the data sequence **c**~**a** is known to the receiver. On the other hand, as already mentioned in the introductory section, via an NDA approach, we are in a position to take arbitrarily long portions of the payload data without impacting the spectral efficiency, as would be the case for a DA solution.

Regarding the computation of the CRLB, the likelihood function in (8) must be averaged first with respect to **c** before deriving the FIM elements [28]. Consequently, the related LLF is determined by

(33) $$\Lambda (y;u)=\log E_{c}[\mathrm{Pr}(y|c;u)]=\log\left(\frac{1}{M^{L}}\sum_{c\in A^{L}}e^{\Lambda (y|c;u)}\right)$$

where $A^{L}$ denotes the *L*-dimensional space spanned by the *M*-ary PAM alphabet. Of course, the computational complexity of (33) is in the order of $O(M^{L})$, i.e., even for smaller values of *L,* the evaluation of (33) is out of scope. Therefore, we will concentrate in the current paper on the derivation of the asymptotic Cramer–Rao lower bound (ACRLB), which applies only to lower SNR values but is achievable in closed form. To this end, the likelihood function is rewritten as

(34) $$\mathrm{Pr}(y|c;u)=e^{\Lambda (y|c;u)}=P_{n}^{-L}e^{-\Phi_{1}(y;u)+\Phi_{2}(y|c;u)}$$

where

(35) $$\Phi_{1}(y;u)=\frac{y^{T}\Psi \;y}{4(1+\alpha )P_{n}}$$

and

(36) $$\Phi_{2}(y|c;u)=\frac{2\sqrt{\rho_{s}P_{n}}\;y^{T}\Psi \;c-\rho_{s}P_{n}\;c^{T}\Psi \;c}{4(1+\alpha )P_{n}}$$

By series expansion of (36), we obtain for smaller SNR values:

(37) $$\mathrm{Pr}(y|c;u)={\left.P_{n}^{-L}e^{-\Phi_{1}(y;u)}\sum_{k=0}^{\infty }\frac{\Phi_{2}^{k}(y|c;u)}{k!}\;\right|}_{\rho_{s}\ll 1}\approx P_{n}^{-L}e^{-\Phi_{1}(y;u)}[1+\Phi_{2}(y|c;u)]$$

and averaging with respect to **c** yields

(38) $$\mathrm{Pr}(y;u)={\left.E_{c}[\mathrm{Pr}(y|c;u)]\right|}_{\rho_{s}\ll 1}\approx P_{n}^{-L}e^{-\Phi_{1}(y;u)}[1+\Phi_{2}(y;u)]$$

where $\Phi_{2}(y;u)=E_{c}[\Phi_{2}(y|c;u)]$. As a result, the corresponding LLF is approximated by

(39) $$\begin{array}{ll} \Lambda (y;u) & =\log\mathrm{Pr}(y;u)\\ & \approx -L\log P_{n}-\Phi_{1}(y;u)+\log[1+\Phi_{2}(y;u)]\\ & \approx -L\log P_{n}-\Phi_{1}(y;u)+\Phi_{2}(y;u)\; \end{array}$$

In computing the second-order derivatives with respect to *ρ_s_* and *P_n_*, in the next step we arrive at

(40) $$\frac{\partial^{2}\Lambda (y;u)}{\partial \rho_{s}^{2}}=\frac{\partial^{2}\Phi_{2}(y;u)}{\partial \rho_{s}^{2}}=-\frac{S_{1}(y)}{8(1+\alpha )\sqrt{\rho_{s}^{3}P_{n}}}$$

(41) $$\frac{\partial^{2}\Lambda (y;u)}{\partial P_{n}^{2}}=\frac{L}{P_{n}^{2}}-\frac{\partial^{2}\Phi_{1}(y;u)}{\partial P_{n}^{2}}+\frac{\partial^{2}\Phi_{2}(y;u)}{\partial P_{n}^{2}}=\frac{L}{P_{n}^{2}}-\frac{S_{2}(y)}{2(1+\alpha )P_{n}^{3}}+\frac{3\sqrt{\rho_{s}P_{n}}S_{1}(y)}{8(1+\alpha )P_{n}^{3}}$$

(42) $$\frac{\partial^{2}\Lambda (y;u)}{\partial \rho_{s}\partial P_{n}}=\frac{\partial^{2}\Phi_{2}(y;u)}{\partial \rho_{s}\partial P_{n}}=-\frac{S_{1}(y)}{8(1+\alpha )\sqrt{\rho_{s}P_{n}^{3}}}$$

where $S_{1}(y)=E_{c}[y^{T}\Psi \;\;c]$ and $S_{2}(y)=y^{T}\Psi \;\;y$. The related FIM elements are achieved by substituting **y** into (40)–(42). The results are then averaged with respect to **y**, i.e., with respect to data and noise sequence, **c** and **ν**, so that we have

(43) $$J_{11}=-E_{y}\left[\frac{\partial^{2}\Lambda (y;u)}{\partial \rho_{s}^{2}}\right]=\frac{S_{1}}{8(1+\alpha )\sqrt{\rho_{s}^{3}P_{n}}}$$

(44) $$J_{22}=-E_{y}\left[\frac{\partial^{2}\Lambda (y;u)}{\partial P_{n}^{2}}\right]=-\frac{L}{P_{n}^{2}}+\frac{S_{2}}{2(1+\alpha )P_{n}^{3}}-\frac{3\sqrt{\rho_{s}P_{n}}S_{1}}{8(1+\alpha )P_{n}^{3}}$$

(45) $$J_{12}=-E_{y}\left[\frac{\partial^{2}\Lambda (y;u)}{\partial \rho_{s}\partial P_{n}}\right]=\frac{S_{1}}{8(1+\alpha )\sqrt{\rho_{s}P_{n}^{3}}}$$

where $S_{1}=E_{y}[S_{1}(y)]$ and $S_{2}=E_{y}[S_{2}(y)]$ provided by (A3) and (A11) in Appendix A. With this in mind, the ACRLB is obtained after some lengthy but straightforward manipulations as

(46) $$\begin{array}{ll} \mathrm{ACRLB}(\rho_{s}) & =\frac{J_{22}}{J_{11}J_{22}-J_{12}^{2}}\\ & \;=\frac{8(1+\alpha )\rho_{s}}{L\mu_{a}^{2}({\bar{\Psi }}_{0}+2{\bar{\Psi }}_{1})\left(1-\frac{\mu_{a}^{2}({\bar{\Psi }}_{0}+2{\bar{\Psi }}_{1})\rho_{s}}{8(1+\alpha )(2{\bar{\Psi }}_{2}-1)+(4{\bar{\Psi }}_{0}-3\mu_{a}^{2}{\bar{\Psi }}_{0}+2\mu_{a}^{2}{\bar{\Psi }}_{1})\rho_{s}}\right)}\; \end{array}$$

Normalized by $\rho_{s}^{2}$, the relationship is for lower SNR values, approximated by

(47) $$\mathrm{NACRLB}(\rho_{s})={\left.\frac{\mathrm{ACRLB}(\rho_{s})}{\rho_{s}^{2}}\right|}_{\rho_{s}\ll 1}\approx \frac{8(1+\alpha )}{L\mu_{a}^{2}\rho_{s}({\bar{\Psi }}_{0}+2{\bar{\Psi }}_{1})}$$

### 4.2. Moment-Based Estimator

Regarding RF systems, moment-based (MB) estimators are well established when it comes to SNR estimation [30,31]. One of the main reasons behind this is that they are often very simple from a computational point of view, e.g., the algorithm based on second- and fourth-order moments frequently employed in the RF context, but they are also very powerful in that their error performance approaches the CRLB. However, the latter is only true for constant-modulus constellations like PSK, whereas for non-constant constellations like QAM, they exhibit a significant degradation, which could be mitigated by resorting to higher-order moments as shown in [32,33]. A particular simple solution is available if the MB principle is applied to the dual-filter framework in Figure 1, as shown in the sequel.

Computing the first-order moment of the samples at the output of *q*(*t*), we have

(48) $$M_{1}=E[z_{k}]=A\;E[a_{k}]+E[n_{k}]=A\;\mu_{a}$$

whereas the second-order moment of the samples at the output of $\dot{q}(t)$ is given by

(49) $$M_{2}=E[{\dot{z}}_{k}^{2}]=E[{\dot{n}}_{k}^{2}]=\frac{8\;\pi^{2}{\left(1+\alpha \right)}^{3}}{3}P_{n}$$

Of course, in a practical scenario, the moments in (48) and (49) are replaced by finite sums according to

(50) $${\hat{M}}_{1}=\hat{A}\;\mu_{a}=\frac{1}{L}\sum_{k=0}^{L-1}z_{k}$$

and

(51) $${\hat{M}}_{2}=\frac{8\;\pi^{2}{\left(1+\alpha \right)}^{3}}{3}{\hat{P}}_{n}=\frac{1}{L}\sum_{k=0}^{L-1}{\dot{z}}_{k}^{2}$$

so that the related SNR estimate is furnished as

(52) $${\hat{\rho }}_{s}=\frac{{\hat{A}}^{2}}{{\hat{P}}_{n}}=\frac{8\;\pi^{2}{\left(1+\alpha \right)}^{3}}{3\mu_{a}^{2}}\frac{{\hat{M}}_{1}^{2}}{{\hat{M}}_{2}}$$

For convenient reasons, the MB algorithm is summarized as follows (Algorithm 2):
**Algorithm 2**: MB estimator.      **Initialization**           Collection of *L* filter outputs: $z_{k},\;\;{\dot{z}}_{k}$      **Computation**           Modified first-order moment:
${\hat{M}}_{a}\leftarrow \sum_{k=0}^{L-1}z_{k}$           Modified second-order moment: ${\hat{M}}_{b}\leftarrow \sum_{k=0}^{L-1}{\dot{z}}_{k}^{2}$      **Output**           SNR estimate:
${\hat{\rho }}_{s}\leftarrow \frac{8\;\pi^{2}{\left(1+\alpha \right)}^{3}}{3\mu_{a}^{2}}\frac{{\hat{M}}_{a}^{2}}{{\hat{M}}_{b}}$

Since the MB algorithm is reduced to the calculation of first- and second-order moments, it is obvious that the computational complexity of the related SNR estimate is just in the order of O(L) real-valued additions and multiplications.

## 5. Numerical Results

**Figure 2:**
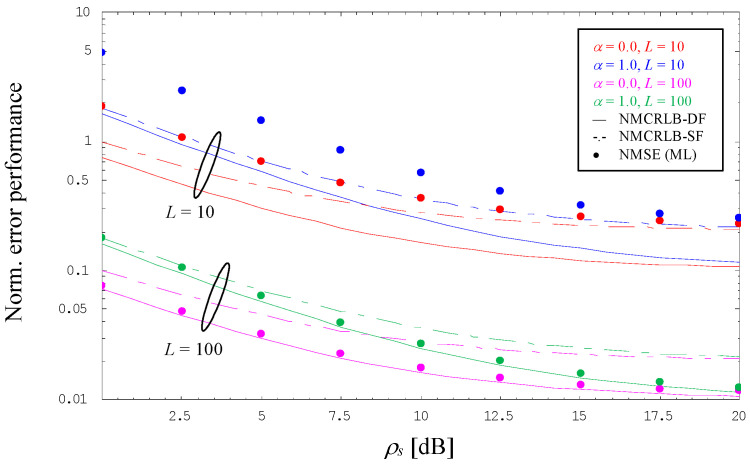
Normalized mean square error for data-aided SNR estimation (4-PAM).

Using a 4-PAM signal operated with no excess bandwidth, i.e., *α* = 0, as well as its maximum value specified by *α* = 1, Figure 2 illustrates the evolution of the error performance, normalized by $\rho_{s}^{2}$, as a function of $\rho_{s}=\gamma_{s}/P_{0}^{2}$ in dB for DA estimation of the SNR. For this purpose, a rather small value for the length of the pilot sequence, embodied by *L* = 10, and a larger one with *L* = 100 have been assumed. For the dual-filter solution analyzed in the current paper, we can see that the normalized MCRLB in (27)—in the diagram denoted by NMCRLB-DF and shown by solid lines—exhibits for $\rho_{s}\to \infty$ the same value irrespective of the selected roll-off factor, which is proportional to 1/*L*, whereas for $\rho_{s}\ll 1$ this proportionality is given by (1 + *α*)/*L*. For comparison purposes, the diagram also includes the normalized limit in the case of a single filter approach [11], which is denoted by NMCRLB-SF and shown in dashed-dotted style. By detailed inspection, we observe that the single-filter results are for $\rho_{s}\to \infty$ approximately twice as large as those achieved with the dual-filter method; this is explained by the fact that, with the output of the parallel filter, twice as many samples are available for estimation purposes.

**Figure 3:**
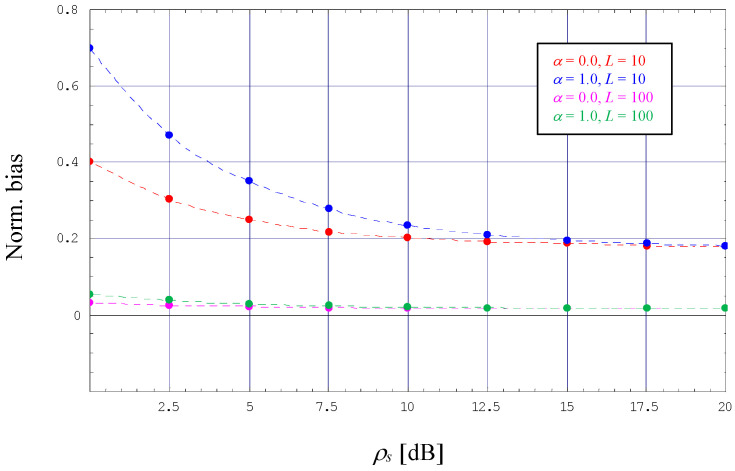
Normalized bias for data-aided ML SNR estimation (4-PAM).

However, with *L* = 10, we also observe that the ML estimator developed in Section 3.3 performs a normalized mean square error (NMSE) with a non-negligible amount of degradation, i.e., although evolving in parallel to the NMCRLB-DF, the difference to the theoretical limit is considerable. The reason for this deficiency is a significant bias effect, which is detailed in Figure 3 (the dotted lines are not related to any analytical work; they are just obtained by cubic interpolation of the simulation results embodied by solid dots in different styles). Nevertheless, the diagram also demonstrates that the bias might be reduced significantly when we increase the length of our pilot sequence to *L* = 100. This is confirmed by Figure 2, where the NMSE generated by the corresponding ML estimator is now very close to NMCRLB-DF.

**Figure 4:**
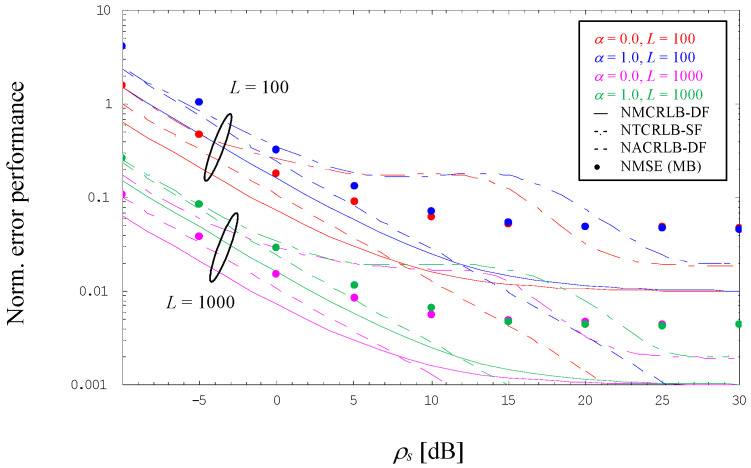
Normalized mean square error for non-data-aided SNR estimation (4-PAM).

Using again a 4-PAM constellation applied to *α* = 0 and 1, Figure 4 visualizes the normalized error performance for NDA estimation of the SNR, but now for larger observation lengths, i.e., *L* = 100 and 1000, as is typical for an NDA situation. For comparison purposes, the diagram includes the evolution of NMCRLB-DF given by (27), in the diagram shown by solid lines, but also that of the normalized true CRLB for NDA estimation of the SNR assuming a single filter receiver (NTRCLB-SF), illustrated in dashed-dotted style, which has been derived in [10]. For $\rho_{s}\to \infty$, we see that the single-filter limit is again twice as large as NMCRLB-DF, irrespective of the chosen excess bandwidth.

The most interesting phenomenon in this respect is that NTRCLB-SF deviates significantly from NMCRLB-DF in the medium SNR range. However, the effect is well known from the open literature when it comes to non-data-aided SNR estimation of non-constant modulus constellations, like PAM or QAM schemes [10,34]. And it is in particular this SNR domain where the simple MB algorithm developed in Section 4.2 is considerably better than the single-filter bound. On the other hand, for $\rho_{s}\to \infty$ the normalized MSE of the MB algorithm is approximately two times larger than NTCRLB-SF, irrespective of the chosen value of *α*.

**Figure 5:**
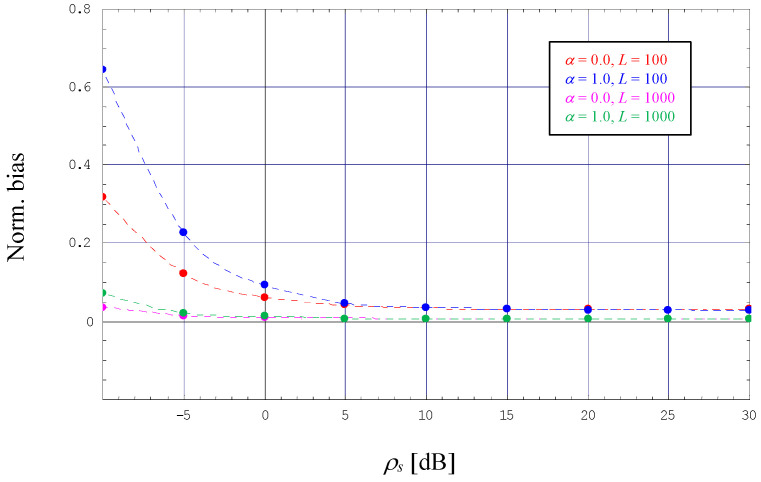
Normalized bias for non-data-aided MB SNR estimation (4-PAM).

For $\rho_{s}\ll 1$ and *L* = 1000, the error performance of MB estimates is close to the normalized asymptotic CRLB for the dual-filter solution expressed by (47), in Figure 4 denoted by NACRLB-DF and depicted in dashed style. In this context, it is to be recalled that—similar to NMCRLB-DF—the asymptotic variant is also proportional to (1 + *α*)/*L*. Not surprisingly, a non-negligible discrepancy is observed for smaller observation intervals, as exemplified by *L* = 100, the reason for which is a non-negligible bias effect exhibited by the MB algorithm. This sort of degradation vanishes more and more with increasing values of *L,* as confirmed by Figure 5.

Comparing the DA and NDA methods, we can see that at least ten times the value of *L* is necessary for the NDA solution, i.e., *L* ≥ 1000, to reduce the bias effect to an amount such that it does not have an impact on the error performance. In this respect, it is to be mentioned again that this does not affect the spectral efficiency of the communication system as such because no pilot data are needed for MB algorithms.

## 6. Conclusions

The major motivation of the current paper were some drawbacks and shortcomings of algorithms investigated in a previous work about NDA SNR estimation for bandlimited optical intensity links. For this reason, the implementation of a second receiver filter with particular properties has been suggested, which is operated in parallel to the receiver filter normally used in this context. Although not intended at the beginning, it could be shown that the approach might be applied to DA scenarios as well. In this respect, an ML algorithm has been developed whose error performance is for larger pilot sequences close to the CRLB as the theoretical limit; smaller pilot sequences exhibit some degradation, which is explained by a non-negligible bias effect.

For the NDA approach, it turned out that the computation of the CRLB would be out of scope even for smaller observation lengths. Therefore, the focus was on the derivation of an asymptotic version of the CRLB, which applies to lower SNR values where a closed-form solution could be achieved. In addition to this, a moment-based SNR estimator has been obtained, which is very simple from a complexity point of view and performs in the medium SNR range significantly better than the CRLB developed for a single-filter solution.

Finally, it is to be mentioned that the ML and MB algorithms have been verified with other parameter setups as well, in particular with symbol constellations other than 4-PAM, but the observations were in principle the same as those made with respect to 4-PAM.

## Data Availability

Data are available from the author upon mail request.

## Appendix A

In this appendix, we detail the expected operations needed for the computation of the asymptotic CRLB in Section 4.1. First, we focus on the averaging of $S_{1}(y)$ with respect to **c**~**a**, i.e., $S_{1}(y)=E_{c}[y^{T}\Psi \;\;c]$. By detailed inspection of the signal model in (7), we have

(A1) $$y^{T}\Psi \;\;c=\sum_{i=0}^{L-1}\sum_{k=0}^{L-1}z_{i\;}\psi_{11,ik}a_{k}+\sum_{i=0}^{L-1}\sum_{k=0}^{L-1}{\dot{z}}_{i\;}\psi_{21,ik}a_{k}$$

such that the averaging with respect to *a_k_* leads to

(A2) $$S_{1}(y)=\mu_{a}\sum_{i=0}^{L-1}\sum_{k=0}^{L-1}z_{i\;}\psi_{11,ik}+\mu_{a}\sum_{i=0}^{L-1}\sum_{k=0}^{L-1}{\dot{z}}_{i}\;\psi_{21,}{}_{ik}$$

In the next step, in order to obtain $S_{1}=E_{y}[S_{1}(y)]$, we have to perform the averaging procedure with respect to $z_{i}=A\;a_{i}+n_{i}$ = $\sqrt{\rho_{s}P_{n}}\;a_{i}+n_{i}$ as well as ${\dot{z}}_{i}={\dot{n}}_{i}$. By taking into account that these are zero-mean processes, we get

(A3) $$\begin{array}{ll} S_{1} & =\mu_{a}\sum_{i=0}^{L-1}\sum_{k=0}^{L-1}E[z_{i}]\;\psi_{11,ik}+\mu_{a}\sum_{i=0}^{L-1}\sum_{k=0}^{L-1}E[{\dot{z}}_{i}]\;\psi_{21,}{}_{ik}\\ & =\mu_{a}^{2}\sqrt{\rho_{s}P_{n}}\;\sum_{i=0}^{L-1}\sum_{k=0}^{L-1}\psi_{11,ik}\\ & =L\mu_{a}^{2}\sqrt{\rho_{s}P_{n}}({\bar{\Psi }}_{0}+2{\bar{\Psi }}_{1}) \end{array}$$

where the last line is achieved by considering the sums in (24). Furthermore, by application of the signal model to $S_{2}(y)=y^{T}\Psi \;\;y$, we have

(A4) $$y^{T}\Psi \;\;y=\sum_{i=0}^{L-1}\sum_{k=0}^{L-1}\left(z_{i\;}\psi_{11,ik}z_{k}+z_{i\;}\psi_{12,ik}{\dot{z}}_{k}+{\dot{z}}_{i\;}\psi_{21,ik}z_{k}+{\dot{z}}_{i\;}\psi_{22,ik}{\dot{z}}_{k}\right)$$

Averaging this relationship with respect to *z_i_* and *z_k_* yields

(A5) $$\begin{array}{ll} S_{2} & =E_{y}[S_{2}(y)]\\ & =\sum_{i=0}^{L-1}\sum_{k=0}^{L-1}\left\{E[z_{i\;}z_{k}]\;\psi_{11,ik}+E[z_{i\;}{\dot{z}}_{k}]\;\psi_{12,ik}+E[{\dot{z}}_{i\;}z_{k}]\;\psi_{21,ik}+E[{\dot{z}}_{i\;}{\dot{z}}_{k}]\;\psi_{22,ik}\right\}\; \end{array}$$

In this context, one has to keep in mind that

(A6) $$\begin{array}{ll} E[z_{i}z_{k}] & =\rho_{s}P_{n}\;E[a_{i}a_{k}]+2\sqrt{\rho_{s}P_{n}}E[a_{i}n_{k}]+E[n_{i}n_{k}]\\ & =\left\{\begin{array}{l} \rho_{s}P_{n}\;+2(1+\alpha )\omega_{11,ii}P_{n}\;,\;\;\;i=k\\ \mu_{a}^{2}\rho_{s}P_{n}+2(1+\alpha )\;\omega_{11,ik}P_{n}\;,\;\;\;i\neq k \end{array}\right. \end{array}$$

(A7) $$E[z_{i}{\dot{z}}_{k}]=\sqrt{\rho_{s}P_{n}}E[a_{i}{\dot{n}}_{k}]+E[n_{i}{\dot{n}}_{k}]=2(1+\alpha )\;\omega_{12,ik}P_{n}$$

(A8) $$E[{\dot{z}}_{i}z_{k}]=\sqrt{\rho_{s}P_{n}}E[{\dot{n}}_{i}a_{k}]+E[{\dot{n}}_{i}n_{k}]=2(1+\alpha )\;\omega_{21,ik}P_{n}$$

(A9) $$E[{\dot{z}}_{i}{\dot{z}}_{k}]=E[{\dot{n}}_{i}{\dot{n}}_{k}]=2(1+\alpha )\;\omega_{22,ik}P_{n}$$

Therefore,

(A10) $$\begin{array}{c} S_{2}=\rho_{s}P_{n}\;\sum_{i=0}^{L-1}\psi_{11,ii}+\mu_{a}^{2}\rho_{s}P_{n}\;\sum_{i=0}^{L-1}\sum_{k=0,k\neq i}^{L-1}\psi_{11,ik}+2(1+\alpha )P_{n}\;\sum_{i=0}^{L-1}\sum_{k=0}^{L-1}\omega_{11,ik\;}\psi_{11,ik}\\ +\;\;2(1+\alpha )P_{n}\sum_{i=0}^{L-1}\sum_{k=0}^{L-1}\left(\omega_{12,ik}\;\psi_{12,ik}+\omega_{21,ik}\;\psi_{21,ik}+\omega_{22,ik}\;\psi_{22,ik}\right)\; \end{array}$$

and by taking into account (24) and (26), we finally get

(A11) $$S_{2}=L\;[\rho_{s}P_{n}\;{\bar{\Psi }}_{0}+2\mu_{a}^{2}\rho_{s}P_{n}\;{\bar{\Psi }}_{1}+4(1+\alpha )P_{n}\;{\bar{\Psi }}_{2}]$$
